# Chimeric antigen receptor T-cell therapy following autologous transplantation for secondary central nervous system lymphoma: A case report

**DOI:** 10.1097/MD.0000000000027733

**Published:** 2021-11-05

**Authors:** Yu Yagi, Yusuke Kanemasa, An Ohigashi, Yuka Morita, Taichi Tamura, Shohei Nakamura, Yuki Otsuka, Yuya Kishida, Akihiko Kageyama, Takuya Shimizuguchi, Takashi Toya, Hiroaki Shimizu, Yuho Najima, Takeshi Kobayashi, Kyoko Haraguchi, Noriko Doki, Yoshiki Okuyama, Yasushi Omuro, Tatsu Shimoyama

**Affiliations:** a Department of Medical Oncology, Tokyo Metropolitan Cancer and Infectious Diseases Center, Komagome Hospital, Tokyo, Japan; b Hematology Division, Tokyo Metropolitan Cancer and Infectious Diseases Center, Komagome Hospital, Tokyo, Japan; c Department of Radiation Oncology, Tokyo Metropolitan Cancer and Infectious Diseases Center, Komagome Hospital, Tokyo, Japan; d Division of Transfusion and Cell Therapy, Tokyo Metropolitan Cancer and Infectious Diseases Center, Komagome Hospital, Tokyo, Japan.

**Keywords:** autologous stem cell transplantation, case report, central nervous system relapse, chimeric antigen receptor T-cell therapy, intravascular large B-cell lymphoma

## Abstract

**Rationale:**

Chimeric antigen receptor (CAR) T-cell therapy is effective in treating relapsed and refractory B-cell non-Hodgkin lymphoma. However, because of the mortality risk associated with immune effector cell-associated neurotoxicity syndrome and pseudoprogression, patients with central nervous system (CNS) involvement are less likely to receive CAR T-cell therapy.

**Patients concerns:**

We report a case of a 61-year-old, male patient with intravascular large B-cell lymphoma who suffered a CNS relapse after standard chemotherapy.

**Diagnosis:**

A diagnosis of intravascular large B-cell lymphoma with CNS involvement was made.

**Interventions:**

We treated the patient using CAR T-cell therapy following a conditioning regimen consisting of thiotepa and busulfan and autologous stem cell transplantation. Although he experienced grade 1 cytokine release syndrome, no other serious adverse events, such as immune effector cell-associated neurotoxicity syndrome or pseudoprogression, were observed.

**Outcomes:**

The patient achieved complete remission after the CAR T-cell infusion.

**Lessons:**

CAR T-cell therapy following autologous stem cell transplantation is a viable option for relapsed/refractory lymphoma with CNS infiltration. Further clinical studies are warranted to verify its safety and efficacy.

## 1 Introduction

The therapeutic efficacy of chimeric antigen receptor (CAR) T-cell therapy has revolutionized the treatment of relapsed/refractory (R/R) B-cell non-Hodgkin lymphoma.^[1]^ However, CAR T-cell therapy can have potentially severe side effects, such as cytokine release syndrome (CRS) and CAR T-cell therapy-related immune effector cell-associated neurotoxicity syndrome (ICANS).^[2]^ The incidence of CRS and ICANS in patients with hematological malignancies is significantly higher than in those with other solid malignancies.^[3]^ Due to the mortality risk associated with ICANS, patients with central nervous system (CNS) involvement are excluded from almost all clinical trials of CAR T-cell therapy. Although there are some reports of the application of CD19-targeted CAR T-cells in CNS lymphoma treatment,^[4–6]^ there are no reports of an increased incidence of ICANS in patients with CNS lymphoma so far. The efficacy and safety of CAR T-cell therapy in patients with CNS involvement have not been established yet due to the rarity of these patients.

Herein, we reported a case of a CNS relapse of intravascular large B-cell lymphoma (IVLBCL) successfully treated with autologous stem cell transplantation (ASCT) followed by CAR T-cell therapy. This study was approved by the Ethics Committee of Tokyo Metropolitan Cancer and Infectious Diseases Center, Komagome Hospital. Written informed consent was obtained from the patient for publication of this case report.

## 2 Case report

**Figure 1:**
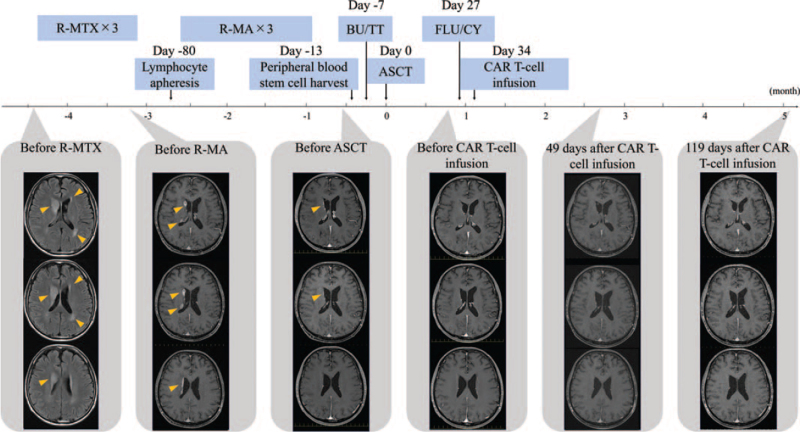
Clinical course: The patient received R-MTX (rituximab and methotrexate) and R-MA (rituximab, methotrexate, and cytarabine) after a central nervous system (CNS) relapse. He received BuTT (busulfan and thiotepa) as the conditioning regimen followed by ASCT (autologous stem cell transplantation). Posttransplantation, he received lymphodepleting chemotherapy consisting of FLU/CY (fludarabine and cyclophosphamide) and an infusion of anti-CD19 CAR (chimeric antigen receptor) T-cells. Brain MRI after the CAR-T cell infusion demonstrated resolution of the CNS lesions, indicating a complete response. The arrowheads show the presence of lymphoma lesions in the brain. MRI = magnetic resonance imaging.

**Figure 2:**
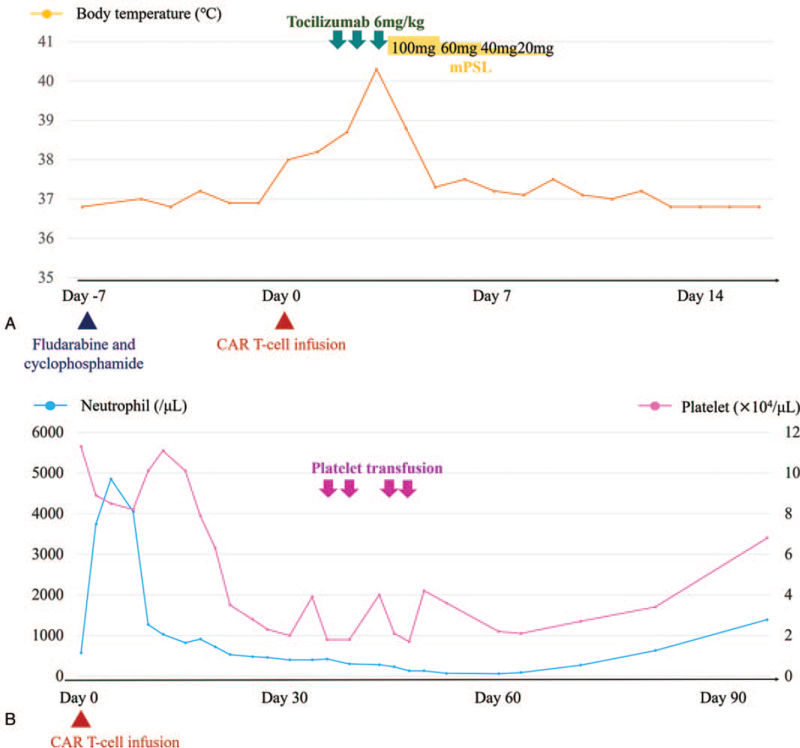
Clinical course after CAR T-cell infusion: Fever ≥ 38°C without hypotension or hypoxia developed after CAR (chimeric antigen receptor) T-cell infusion, corresponding to grade 1 cytokine release syndrome. The patient was treated with tocilizumab and methylprednisolone (mPSL) (A). Grade 4 neutropenia and thrombocytopenia developed after CAR T-cell infusion (B).

A previously healthy, 61-year-old, male patient presented with fever and dyspnea. On admission, a blood test revealed a marked elevation of lactate dehydrogenase (1450 U/L normal: 106–211 U/L) and interleukin 2 receptor (8800 U/mL normal: 122–496 U/mL). Computed tomography revealed consolidation in the upper lobe. IVLBCL was diagnosed based on an analysis of bone marrow, lung biopsy, and skin biopsy specimens. Brain magnetic resonance imaging (MRI) revealed no abnormalities, and the patient was not considered to have lymphoma with CNS involvement. He achieved complete remission (CR) after 6 cycles of cyclophosphamide, doxorubicin, vincristine, and prednisone and 8 cycles of rituximab plus 2 cycles of high-dose methotrexate (HD-MTX). One month later, he experienced a CNS relapse with brain lesions detected by positron emission tomography and computed tomography. MRI revealed a 25 mm × 20 mm × 34 mm mass in the right basal ganglia and other smaller lesions in the brain (Fig. 1). No lymphoma cells were found in cerebrospinal fluid, nor were any lesions detected outside the CNS. Owing to the difficulty of curing a CNS relapse by salvage chemotherapy even with ASCT, the addition of CAR T-cell therapy after ASCT was planned. He underwent 3 cycles of HD-MTX and rituximab followed by 3 cycles of HD-MTX/cytarabine. Lymphocyte apheresis for CAR T-cell manufacturing was performed during the salvage chemotherapy. Subsequent MRI showed a partial response. A peripheral blood stem cell harvest was performed, and the patient received a conditioning regimen consisting of BuTT (thiotepa 5 mg/kg on days -7 to -6, busulfan 3.2 mg/kg on days -5 to -4) and ASCT on day 0. Although MRI after the ASCT was unable to detect the residual lesions, the patient was still considered to have residual CNS disease. He received lymphodepleting chemotherapy consisting of fludarabine 25 mg/m^2^/day and cyclophosphamide 250 mg/m^2^/day on days -7 to -5 and an infusion of anti-CD19 CAR T-cells (tisagenlecleucel) on day 0. Four hours after the CAR T-cell infusion, a fever, which failed to respond to broad-spectrum antibiotics or supportive treatment, developed without hypotension or hypoxia, leading to the diagnosis of grade 1 CRS. Although the patient was treated with tocilizumab 8 mg/kg/day on days 2 and 3, the symptoms continued. Finally, he was given methylprednisolone 2 mg/kg/day from day 3, and his temperature normalized by day 5. Thereafter the methylprednisolone was tapered, then stopped on day 8. No neurotoxic events were observed during the follow-up period. Grade 4 neutropenia developed from day 23 and improved by day 78. No serious infections were observed. Grade 4 thrombocytopenia developed from day 36 and improved by day 47 (Fig. 2). One month after the CAR T-cell infusion, brain MRI showed a complete absence of brain lesions, indicating a CR (Fig. 1). Eight months after the CAR T-cell infusion, the patient remains in CR.

## 3 Discussion

The patient in the present study had R/R B-cell non-Hodgkin lymphoma with a CNS relapse and failed to achieve a CR with salvage chemotherapy, high-dose chemotherapy (HDC) or ASCT. After CAR T-cell therapy, he finally achieved durable CR without any severe adverse events, such as ICANS. To the best of our knowledge, the present report is the first to demonstrate the safety and efficacy of CAR T-cell therapy following ASCT in patients with CNS involvement.

Previous studies reported the risk of CNS relapse in diffuse large B-cell lymphoma (DLBCL) to be approximately 5%.^[7–9]^ IVLBCL is characterized by clinically aggressive behavior and a high rate of CNS involvement at diagnosis. Several, previous studies have examined IVLBCL patients with a CNS relapse, and a retrospective study reported that the risk of CNS recurrence at 3 years was 25% among 82 patients without CNS involvement at the initial diagnosis.^[10–13]^

The incidence of CNS involvement secondary to DLBCL is considerably higher in patients with certain, high-risk, clinical features at diagnosis. Multivariate risk models, such as CNS-IPI, are effective in predicting the risk of CNS recurrence.^[14]^ Identifying patients with a high risk of CNS relapse allows us to offer appropriate prophylaxis during the first line therapy. Intravenous and intrathecal prophylaxis is most frequently used, but the evidence is equivocal as to its effectiveness.^[15]^ Intravenous HD-MTX can be safely administered in combination with standard chemoimmunotherapy and decreases the risk of CNS recurrence in patients with high-risk DLBCL. However, there is currently no standard form of CNS-prophylaxis.^[15]^

The prognosis of patients with a CNS relapse is still poor, and there is as of yet no consensus on the optimal salvage treatment. The median overall survival after CNS relapse is reportedly about 7 months.^[16,17]^ The data supporting the efficacy of HDC and ASCT in patients with DLBCL with secondary CNS lymphoma are limited although some studies have shown favorable survival outcomes (the median DFS and overall survival for patients with CR at ASCT was 36 and 33 months, respectively).^[18–21]^ The optimal HDC regimen for secondary CNS lymphoma is also unknown. Conditioning regimens containing agents that can effectively penetrate the blood-brain barrier, such as BuTT, are reportedly associated with promising disease control for relapsed CNS lymphoma. Some retrospective analyses reported an association between the thiotepa/busulfan-based conditioning therapy with ASCT for secondary CNS lymphoma and progression-free survival at years 2 and 3 of 76.1% to 93%, respectively.^[18,19]^ Despite the use of the HDC regimen and ASCT in the treatment of CNS lesions, a complete cure for secondary CNS lymphoma is still elusive. CAR T-cell therapy may therefore augment these therapies to improve the possibility of CR.

The occurrence of lethal neurotoxicity after CAR T-cell therapy prevents most patients with a CNS lesion from receiving this treatment. Abramson et al^[4]^ reported the first case of CNS lymphoma treated with CAR T-cell therapy, which induced CR without ICANS. A retrospective analysis of tisagenlecleucel for the treatment of R/R B-cell non-Hodgkin lymphoma with secondary CNS involvement showed that none of the patients experienced CAR T-cell-related neurotoxicity.^[5]^ In addition, the rate of ICNAS did not increase in 5 patients treated with axicabtagene ciloleucel for R/R NHL with secondary CNS involvement.^[22]^

The pathogenesis of ICANS is not completely understood but may be related to cytokine-mediated toxicity resulting from endothelial dysfunction, increased blood-brain barrier permeability, and failure to protect cerebrospinal fluid from high concentrations of systemic cytokines.^[23]^ The most significant risk factor of ICANS is a history of severe CRS. A high tumor burden is also associated with ICANS development.^[24,25]^ In the present case, ICNAS did not occur after CAR T-cell therapy partly because the ASCT after BuTT was able to reduce the tumor burden sufficiently.

Pseudoprogression, a well-defined phenomenon in other immunotherapies for solid tumors, is characterized by a temporary enlargement of the tumors due to immune cell infiltration and is also observed during CAR T-cell therapy. However, this enlargement can cause local compression,^[26]^ and CAR T-cell therapy in the presence of substantial residual disease can be fatal in cases of CNS invasion. Combined with the fact that a durable response to CAR T-cell therapy is associated with a low baseline tumor burden,^[27]^ reducing the tumor burden as much as possible before CAR T-cell therapy, especially in cases of CNS involvement, is crucial.

HDC and ASCT are the current standard of care for R/R DLBCL. However, relapse rates are still high. Some reports demonstrated the feasibility of CAR T-cell therapy a couple of days after ASCT for R/R B-cell non-Hodgkin lymphoma,^[28–30]^ unlike the present case, in which CAR T-cell therapy was performed about 1 month after ASCT. The tumor burden decreases as a result of myeloablative conditioning, and the immunosuppressive microenvironment is attenuated after ASCT. Consecutive administration of ASCT and CAR-T cells eradicates the residual disease and reduces the recurrence rate. In fact, CAR T-cell therapy following ASCT exhibited a higher rate of CR in patients with R/R large B-cell lymphoma than CAR T-cell therapy alone.^[28]^ However, the therapy was associated with a high incidence of reversible neurotoxicity, with 10 of 15 patients in a phase I clinical study experiencing grade 3 to 4 neurotoxicity, which may have been caused by the high-dose conditioning and pegfilgrastim administered immediately before the CAR T-cell infusion.^[29]^ We believe that CAR T-cell therapy after an interval following ASCT can increase the response rate by reducing the tumor volume with less neurotoxicity.

Whole brain radiotherapy (WBRT) for CNS lymphoma prolongs progression-free survival.^[31]^ However, the major impediment to using WBRT is the high risk of late-delayed neurotoxicity, which manifests as progressive leukoencephalopathy with cognitive deterioration often leading to severe dementia and death. In an attempt to minimize neurotoxicity, WBRT is often avoided altogether in the treatment of patients with lymphoma with CNS involvement.^[32]^ CAR T-cell therapy following ASCT may be a viable alternative to WBRT in the treatment of CNS lymphoma.

## 4 Conclusion

CAR T-cell therapy was safely administered after ASCT to a patient with secondary CNS lymphoma. CAR T-cell therapy following ASCT is a promising treatment for patients with lymphoma with CNS involvement. Vigilant follow-up of the present patient and further studies are needed to determine whether CAR T-cell cell therapy following ASCT is indeed a viable therapeutic option for R/R B-cell non-Hodgkin lymphoma with CNS infiltration.

## Acknowledgments

The authors would like to thank the nursing staff at Tokyo Metropolitan Cancer and Infectious Diseases Center, Komagome Hospital for their excellent patient care.

## Author contributions

**Conceptualization:** Tatsu Shimoyama.

**Investigation:** Yu Yagi, Yusuke Kanemasa, An Ohigashi, Yuka Morita, Taichi Tamura, Shohei Nakamura, Yuki Otsuka, Yuya Kishida, Akihiko Kageyama, Takuya Shimizuguchi, Takashi Toya, Hiroaki Shimizu, Yuho Najima, Takeshi Kobayashi, Kyoko Haraguchi, Noriko Doki, Yoshiki Okuyama, Yasushi Omuro.

**Supervision:** Yasushi Omuro, Tatsu Shimoyama.

**Writing – original draft:** Yu Yagi.

**Writing – review & editing:** Yusuke Kanemasa.
